# Functional Characterization of Uracil-DNA Glycosylase from *Staphylococcus aureus*

**DOI:** 10.3390/pathogens15101012

**Published:** 2026-09-24

**Authors:** Arina Koreneva, Albina Sugirova, Bakhtiyar Yakupov, Maria V. Safonkina, Anton V. Endutkin, Dmitry O. Zharkov, Murat Saparbaev, Sailau Abeldenov, Aigerim Turgimbayeva

**Affiliations:** 1 National Center for Biotechnology, Astana 010000, Kazakhstan; 2 Institute of Animal Science and Veterinary, Saken Seifullin Kazakh Agrotechnical University, Astana 010000, Kazakhstan; 3 Department of Natural Sciences, Novosibirsk State University, Novosibirsk 630090, Russia; 4 Knorre Institute of Chemical Biology and Fundamental Medicine, Siberian Branch of the Russian Academy of Sciences, Novosibirsk 630090, Russia; 5 Team «DNA Repair, ADP-Ribosylation and Cancer», CNRS-UMR9019 Genome Integrity and Cancers, Gustave Roussy, Université Paris-Saclay, 94800 Villejuif, France; 6 Faculty of Biology and Biotechnology, Al-Farabi Kazakh National University, Almaty 050040, Kazakhstan

**Keywords:** base excision repair, DNA repair, DNA glycosylase, uracil excision, *Staphylococcus aureus*, genome stability, enzyme kinetics, AlphaFold 3

## Abstract

**Background/Objectives:** Uracil-DNA glycosylase (UNG) initiates base excision repair by removing uracil from DNA. Although Family 1 UNGs are well characterized in model organisms, the biochemical and functional properties of *Staphylococcus aureus* UNG (SaUNG) is not well characterized. This study aimed to characterize SaUNG. **Methods:** Recombinant wild-type and mutant SaUNG proteins were examined using biochemical and kinetic assays, site-directed mutagenesis, genetic complementation in *Escherichia coli*, and *in vitro* reconstitution of *S. aureus* base excision repair. **Results:** SaUNG exhibited maximal activity at pH 8.0–8.5, 37–45 °C, and 100–150 mM KCl and lacked AP-lyase activity. Catalytic efficiencies (*k_cat_*/*K_m_*) were 1.39 × 10^9^, 3.79 × 10^8^, and 2.93 × 10^8^ M^−1^·min^−1^ for ssU, U:G, and U:A, respectively, establishing the preference ssU > U:G > U:A. H180A retained residual activity, whereas simultaneous substitution of Asp59 and His180 abolished detectable activity. Complete inhibition confirmed conserved biochemical properties of Family 1 UNGs. SaUNG complemented the mutation avoidance defect of an *E. coli* (*ung^−^ mug^−^*) mutant and initiated uracil repair in a reconstituted *S. aureus* base excision repair system. **Conclusions:** SaUNG is a canonical Family 1 UNG with experimentally characterized biochemical properties, catalytic determinants, and biological function in uracil repair.

## 1. Introduction

*Staphylococcus aureus* is one of the most clinically important opportunistic pathogens, colonizing approximately 20–30% of the human population [1]. The bacterium commonly resides in the anterior nares, skin, and upper respiratory tract and, although colonization is often asymptomatic, it represents a major risk factor for subsequent endogenous infections. *S. aureus* infections range from mild skin and soft tissue infections to severe invasive diseases, including osteomyelitis, septic arthritis, bacteremia, pneumonia, and infective endocarditis [2]. Colonization rates may reach up to 80% in specific populations, such as healthcare workers and individuals with chronic medical conditions or compromised immune function [3]. In addition, *S. aureus* is a leading cause of bloodstream infections worldwide. Although effective antimicrobial therapies and source-control strategies are available, *S. aureus* bacteremia remains associated with high mortality, with reported 30-day death rates ranging from 15% to 40% [4]. These features emphasize the importance of identifying the molecular determinants that enable *S. aureus* to survive host defenses, adapt to stressful environments, and maintain its pathogenic potential.

The increasing prevalence of antibiotic-resistant bacterial pathogens highlights the need to identify novel cellular pathways and molecular targets for antimicrobial intervention. Because DNA repair systems are essential for bacterial survival under genotoxic and host-induced stresses, they represent attractive targets for the development of new antibacterial strategies. Among these systems, the base excision repair (BER) pathway plays a central role in the repair of damaged and inappropriate bases, including uracil residues generated by cytosine deamination or dUTP misincorporation, as well as lesions caused by oxidation and alkylation. Given its essential function in maintaining genome integrity, BER and its constituent enzymes have emerged as promising targets for the development of novel antibacterial agents [5,6].

Uracil is one of the most frequently occurring non-canonical bases in DNA and arises through two major mechanisms: spontaneous hydrolytic deamination of cytosine and misincorporation of dUMP during DNA replication due to the utilization of dUTP by replicative DNA polymerases. Deamination of cytosine converts this canonical base into uracil, leading to the formation of a U:G mismatch. If left unrepaired before DNA replication, U:G mismatches may result in G:C → A:T transition mutations. Likewise, replication-associated incorporation of uracil opposite adenine generates U:A pairs that compromise genome integrity and require prompt correction [7]. Failure to eliminate uracil from DNA promotes the accumulation of genetic alterations, particularly G:C → A:T transition mutations. Therefore, efficient surveillance and removal of uracil residues are essential for maintaining genomic stability, with the first line of defense being provided by uracil-DNA glycosylases, which recognize and excise uracil from DNA.

*S. aureus* encodes uracil-DNA glycosylase (SaUNG), a component of the uracil base excision repair machinery that has been identified through genome annotation and studies of its interaction with the endogenous uracil-DNA glycosylase inhibitor SAUGI [8,9]. However, in contrast to the extensively characterized *Escherichia coli* uracil-DNA glycosylase (EcUNG) [10,11,12]. current knowledge of SaUNG remains limited. The ability of the enzyme to cleave uracil-containing DNA has been demonstrated [9,13], the structure of the free protein has been solved (Protein Data Bank ID: 3WDF), and its interactions with proteinaceous inhibitors from *S. aureus* and several bacterial and bacteriophage species have been thoroughly studied at the structural (3WDG, 8AIM, 8AIN) and biochemical levels [8,9,13,14]. Yet the functional properties of SaUNG, its catalytic determinants, and contribution to uracil repair in living cells have not been comprehensively investigated.

A comprehensive evaluation of SaUNG should integrate biochemical, structural, and functional perspectives to define the molecular determinants governing its activity and its contribution to uracil repair in *S. aureus*, thereby providing a broader understanding of uracil base excision repair in this clinically important pathogen. To address this knowledge gap, we performed a comprehensive functional characterization of SaUNG.

## 2. Materials and Methods

### 2.1. Plasmids, Bacterial Strains, Oligonucleotides and Reagents

The plasmids pET-11a (Novagen, Madison, WI, USA), pET-23b (Novagen, Madison, WI, USA) and pBluescript II SK+ (hereafter referred to as pBSK) were used for plasmid construction.

Oligonucleotides used for gene amplification and site-directed mutagenesis were synthesized at the National Center for Biotechnology (Astana, Kazakhstan). Fluorescently labeled and unlabeled uracil-containing DNA substrates, as well as their complementary oligonucleotides used for enzymatic activity assays, were obtained from Lumiprobe RUS LLC (Moscow, Russia). All oligonucleotide sequences used in this study are listed in Table 1.

Restriction enzymes, T4 DNA ligase, and Phusion High-Fidelity DNA Polymerase (Thermo Scientific, Vilnus, Lithuania) were used for gene amplification and cloning. *E. coli* UNG (EcUNG) and UGI used in DNA repair assays were purchased from New England Biolabs (Ipswich, MA, USA). SaNfo and SaDnaN were purified as previously described [15]. For BER reconstitution experiments, EcUNG and phage T4 DNA ligase were purchased from Sibenzyme (Novosibirsk, Russia).

*S. aureus* 29213 (ATCC, Manassas, VA, USA) was used for genomic DNA isolation and subsequent gene cloning. *E. coli* strains DH5α (Thermo Scientific, Vilnius, Lithuania), Rosetta 2 (DE3) (Merck KGaA, Darmstadt, Germany), and MS1022 were obtained from the laboratory collection. Molecular cloning and recombinant protein expression were carried out using *E. coli* DH5α and Rosetta 2 (DE3), respectively. *E. coli* MS1022 strain (as CC102 but *mug-1::Kn^R^ ung-1::Spc^R^*) was used to measure the spontaneous mutation rate [16]. The CC102 strain is a derivative of the P90C strain [*araΔ(lac proB)_XIII_*] and carries a *lacZ* allele on an F′(*lacI^−^*, *Z^−^ proB^+^*) plasmid; therefore, it exhibits a Lac^−^ phenotype. The strain carries an E461G mutation in β-galactosidase, which enables detection of G:C→A:T transitions by measuring the number of Lac^+^ revertants [17]. *E. coli* strains used for molecular cloning and recombinant protein expression were routinely cultivated in LB broth (Condalab, Torrejón de Ardoz, Madrid, Spain). Spontaneous mutation rates were determined using M9 minimal agar medium prepared from M9 Minimal Medium Salts (MP Biomedicals, Solon, OH, USA).

### 2.2. Cloning, Site-Directed Mutagenesis and Purification of SaUNG

Genomic DNA was isolated from *S. aureus* ATCC 29213 using the Genomic DNA Purification Kit (Thermo Scientific, Vilnius, Lithuania). The *ung* gene (657 bp) was amplified using the NdeI-SaUNG fw and BamHI-SaUNG rv primers (Table 1) and cloned into the pET-11a expression vector via the NdeI and BamHI restriction sites. The H180A and D59A/H180A variants were generated by site-directed mutagenesis using the primers listed in Table 1. All constructs were verified by Sanger sequencing. Sequence analysis confirmed complete identity of the cloned *ung* gene with the corresponding reference sequence deposited in GenBank (NC_007795.1; range: 571090–571746).

Recombinant wild-type SaUNG and the SaUNG H180A and D59A/H180A variants were produced by transforming *E. coli* Rosetta 2 (DE3) cells with pET-11a containing the corresponding inserts. Protein expression was induced with 0.5 mM IPTG at OD_600_ = 0.6, followed by incubation at 22 °C with shaking at 100 rpm for 16 h. Cells were harvested by centrifugation at 6000× *g* for 7 min at 4 °C, and the resulting pellet was resuspended in buffer containing 20 mM Tris–HCl (pH 8.0) and 50 mM NaCl supplemented with cOmplete™ Protease Inhibitor Cocktail (Roche, Basel, Switzerland). Cells were treated with lysozyme (3 mg/mL) for 20 min at room temperature and subsequently disrupted by pulsed ultrasonication (50 kHz) on ice using a UCD-1200 ultrasonic cell disrupter (Biobase, Jinan, Shandong, China). The lysate was clarified by centrifugation at 40,000× *g* for 1 h at 4 °C. The supernatant was applied to a HiTrap Heparin HP column (Cytiva, Uppsala, Sweden), and the target protein was eluted using a linear 50–1000 mM NaCl gradient. Purified fractions were further subjected to anion-exchange chromatography on a HiTrap Q HP column (Cytiva, Uppsala, Sweden) using the same NaCl gradient. Fractions containing recombinant proteins were stored at −20 °C in 50% (*v*/*v*) glycerol.

The identity of purified SaUNG was confirmed by MALDI tryptic mapping (91% coverage).

### 2.3. DNA Repair Assays

The 22 bp double-stranded DNA substrates were prepared by annealing the 5′-TAMRA-labeled U-22-TAMRA oligonucleotide with the corresponding complementary oligonucleotide, 22-Compl-A or 22-Compl-G, to generate U:A and U:G duplexes, respectively (Table 1). Annealing was performed in buffer containing 20 mM Tris–HCl (pH 8.0) and 50 mM KCl. The mixtures were heated at 65 °C for 5 min and then gradually cooled to room temperature at a rate of 1 °C min^−1^.

The DNA glycosylase activity of SaUNG was measured in a standard 20 μL reaction mixture containing 20 nM 5′-TAMRA-labeled 22 bp U:G DNA duplex, 10 mM KCl, 20 mM Tris–HCl (pH 8.0), 0.5 mM EDTA, 0.1 mg/mL BSA, 1 mM DTT, and the indicated amount of enzyme. Reactions were carried out at 37 °C for the specified incubation times. The reactions were terminated by adding 2.2 μL of 10 M NaOH, followed by incubation at 65 °C for 10 min to induce alkaline cleavage at the abasic sites generated by uracil excision. After cooling, 0.92 μL of 0.5 M EDTA was added, and the reaction mixtures were neutralized with 2.5 μL of 10.15 M HCl. Reaction products were desalted using homemade spin columns packed with Sephadex G-25 resin (GE Healthcare, Piscataway, NJ, USA) pre-equilibrated with 7.5 M urea. The resulting DNA fragments were separated by denaturing polyacrylamide gel electrophoresis on 20% (*w*/*v*) polyacrylamide gels containing 7.5 M urea in 0.5× TBE buffer. Fluorescent DNA bands were visualized using a ChemiDoc Imaging System (Bio-Rad, Hercules, CA, USA).

The activity of mutant SaUNG variants was evaluated in a standard 20 μL reaction mixture. Wild-type SaUNG was analyzed at final concentrations of 0.25, 0.5, and 1 nM, whereas mutant variants were tested at final concentrations of 20, 50, and 100 nM. Reactions were incubated at 37 °C for 5 min.

The effects of ionic strength, pH, and temperature on SaUNG activity were investigated. The effect of ionic strength was evaluated by varying the final KCl concentration in the standard reaction buffer from 25 to 500 mM. Reactions contained 0.25 nM SaUNG and were incubated at 37 °C for 3 min.

The pH dependence of SaUNG activity was assessed using sodium acetate buffer (pH 4.0 and 5.0), sodium phosphate buffer (pH 6.0), Tris–HCl buffer (pH 7.0, 7.5, 8.0, and 8.5), and glycine–NaOH buffer (pH 9.0). Reactions were performed with 2 nM SaUNG at 37 °C for 5 min.

The effect of temperature on SaUNG activity was examined in the buffer optimized for KCl concentration and pH. Reaction mixtures containing 1 nM SaUNG were incubated at temperatures ranging from 4 to 65 °C for 1 min.

Quantitative analysis of gel images was performed using Image Gauge v4.0 software. Unless otherwise stated, all experiments were performed at least three times independently.

### 2.4. Substrate Specificity Analysis of SaUNG

The substrate specificity of SaUNG was evaluated using a 5′-TAMRA-labeled 22-mer single-stranded uracil-containing oligonucleotide and 5′-TAMRA-labeled 22 bp DNA duplexes containing U:G and U:A base pairs. Reactions were performed in assay buffer containing 20 mM Tris–HCl (pH 8.0), 150 mM KCl, 0.5 mM EDTA, 1 mM DTT, 0.1 mg/mL BSA, and 5 mM MgCl_2_. The reaction mixtures contained 20 nM DNA substrate and 0.5 nM SaUNG. Following incubation at 37 °C for 1 min, reaction products were analyzed by denaturing PAGE as described above.

### 2.5. AP Lyase Activity Assay

To determine whether SaUNG possesses AP lyase activity, reactions were performed using the standard reaction buffer containing 20 nM 5′-TAMRA-labeled 22 bp U:G DNA duplex and SaUNG at the indicated concentration. EcUNG (5 nM; New England Biolabs, Ipswich, MA, USA) was used as a control. Reactions were incubated at 37 °C for 5 min. Following termination of the reactions by heating at 100 °C for 1 min, a subset of samples was treated with 1 M NaOH at 95 °C for 5 min to induce alkaline cleavage, whereas the remaining samples were left untreated. Reaction products were subsequently analyzed by denaturing PAGE as described above.

### 2.6. Inhibition of SaUNG Activity by UGI

The effect of uracil-DNA glycosylase inhibitor (UGI) from *Bacillus subtilis* bacteriophage PBS1 (New England Biolabs, Ipswich, MA, USA) on SaUNG activity was evaluated under the standard assay conditions using 20 nM 5′-TAMRA-labeled 22 bp U:G DNA duplex. EcUNG and SaUNG were used at a final concentration of 0.5 nM. UGI was included at an enzyme-to-inhibitor molar ratio of 1:1. Prior to initiating the reactions, the enzymes were preincubated with UGI on ice for 30 min. The reactions were then carried out at 37 °C for 3 min. Control reactions containing EcUNG or SaUNG in the absence of UGI were performed in parallel. Reaction products were analyzed by denaturing PAGE as described above.

### 2.7. Steady-State Kinetics

To determine the kinetic parameters of SaUNG, initial reaction rates were measured in the standard reaction buffer using U:A, U:G, and single-stranded uracil-containing (ssU) DNA substrates. The substrates were prepared using the 5′-TAMRA-labeled uracil-containing oligonucleotide U-75-TAMRA and the corresponding unlabeled oligonucleotide U-75 (Table 1). The U:A and U:G duplexes were prepared by annealing U-75-TAMRA or U-75 with the corresponding complementary oligonucleotide, 75-Compl-A or 75-Compl-G, respectively, in annealing buffer containing 20 mM Tris–HCl (pH 8.0) and 50 mM KCl. The mixtures were heated at 95 °C for 5 min and then gradually cooled to room temperature at a rate of 1 °C min^−1^.

Total substrate concentrations ranged from 20–500 nM for U:A and U:G and from 20–2000 nM for ssU. In all reactions, the fluorescently labeled substrate was maintained at 20 nM, whereas higher total substrate concentrations were achieved by adding the corresponding unlabeled substrate. For comparison, kinetic parameters of EcUNG were determined under identical experimental conditions. Product formation was quantified during the linear phase of the reaction, and initial velocities (V_0_) were calculated from the amount of product formed per unit time.

### 2.8. Model Building

SaUNG models were generated using AlphaFold 3 [18]. For the DNA-bound models, the duplex was composed of the 5′-TGTFATCTT-3′ (F, (3-hydroxytetrahydrofuran-2-yl)methyl phosphate, a synthetic AP site analog) and 5′-AAAGATAACA-3′ strands [19]. p*K*_a_ values for the titratable groups of SaUNG were calculated by solving the Poisson–Boltzmann equation using H++ v4.0 [20] in a 10 Å box of OPC water (internal dielectric 10, external dielectric 80) at pH 7.5 and 150 mM salt. PyMOL v2.0 (Schrödinger, New York, NY, USA) was used for structural analysis and visualization.

### 2.9. Measurement of Spontaneous Rate of Mutation

The *ung* gene was amplified from the pET-11a/SaUNG plasmid using the BamHI-rbsSaUNG fw and NotI-SaUNG rv primers (Table 1) and cloned into pBluescript II SK(+) at the BamHI and NotI restriction sites. The D59A and H180A substitutions were introduced by site-directed mutagenesis to generate the pBSK/SaUNG D59A/H180A plasmid.

To evaluate the contribution of SaUNG catalytic activity to mutation avoidance *in vivo*, spontaneous mutation rates were determined in the *E. coli* MS1022 (*ung^−^ mug^−^*) strain transformed with the plasmids pBSK, pBSK/EcUNG, pBSK/SaUNG, or pBSK/SaUNG D59A/H180A. For each transformant, five parallel 3 mL cultures were inoculated from independent colonies and grown at 37 °C for 16 h. Cells were harvested by centrifugation (7000× *g*, 4 °C, 7 min) and resuspended in 1 mL of sterile sodium phosphate buffer. To determine the number of Lac^+^ revertants, appropriate dilutions of each culture were plated onto M9 minimal agar medium supplemented with lactose. Mutation rates were determined by the method of the median [21]. The experiment was performed three times independently.

### 2.10. SaPolX Cloning and Purification

The coding sequence for DNA polymerase X from *S. aureus* strain NCTC 8325 (NCBI reference YP_499642.1) was optimized for expression in *E. coli* and synthesized by ShineGene (Shanghai, China). The insert was verified by Sanger sequencing and subcloned into the pET-23b vector at NdeI and XhoI sites, adding a C-terminal His_6_ tag to the full-length protein. *E. coli* T7 Express LysY cells (New England Biolabs, Ipswich, MA, USA) transformed with the expression plasmid were grown overnight at 37 °C in 25 mL of the M9 minimal medium supplemented with 100 μg/mL ampicillin with vigorous shaking. Subsequently, 10 mL of the overnight culture was transferred to 1 L of LB medium. The culture was grown under the same conditions to OD_600_ = 0.7, and protein expression was induced with 0.25 mM IPTG overnight. The cells were collected by centrifugation and frozen at −70 °C until use.

To purify SaPolX, the cells were thawed, resuspended in the lysis buffer (25 mM Tris–HCl (pH 8.0), 500 mM NaCl, 1 mM DTT, 1 mM phenylmethylsulfonyl fluoride, 5% glycerol, 0.01% Nonidet P-40), and disrupted by sonication. The debris was removed by centrifugation, and the supernatant was filtered and loaded onto a 15 mL Ni-NTA agarose column (Qiagen, Venlo, the Netherlands) equilibrated with Buffer A (25 mM HEPES–KOH (pH 8.0), 500 mM NaCl, 5% glycerol) supplemented with 25 mM imidazole. The bound proteins were eluted by a 25–500 mM imidazole gradient in Buffer A. Fractions with the expected electrophoretic mobility were pooled, diluted with Buffer B (25 mM HEPES–KOH (pH 8.0), 1 mM EDTA, 1 mM DTT, 5% glycerol) to a total salt concentration of approximately 100 mM, and loaded onto a 5 mL HiTrap Q HP column (GE Healthcare, Piscataway, NJ, USA) equilibrated with the same buffer. The target protein was eluted using a 100–1000 mM NaCl gradient in Buffer B. Fractions containing the target protein at >90% purity were pooled, dialyzed against the storage buffer (50 mM Tris–HCl (pH 7.5), 400 mM NaCl, 1 mM EDTA, 1 mM DTT, 50% glycerol), and stored at −20 °C until use. The identity of the purified protein was confirmed by MALDI tryptic mapping (53% coverage).

### 2.11. BER Reconstitution Assay

The reaction mixture (10 µL) contained 50 mM Tris–HCl (pH 7.8), 10 mM MgCl_2_, 10 mM DTT, 1 mM dTTP, 1 mM ATP, 100 nM 23 bp U duplex prepared from the fluorescently labeled U-23-Fluo oligonucleotide and the complementary 23-Compl-A oligonucleotide (Table 1), and, where indicated, 100 nM SaUNG, 50 nM SaNfo, 40 nM SaPolX, 100 nM SaDnaN, and 200 U of T4 DNA ligase. Reactions were incubated at 37 °C for 30 min and terminated by adding 1 µL of 1 M NaOH, followed by heating at 95 °C for 3 min and neutralization with an equimolar amount of HCl. Where indicated, the reaction mixture was supplemented with 20 U of EcUNG after neutralization, incubated for an additional 30 min, and subjected to hot alkali treatment again. The reaction mixtures were mixed with half a volume of formamide and analyzed by electrophoresis in a 20% polyacrylamide gel containing 7.2 M urea. The products were visualized using a Typhoon FLA 9500 scanner (GE Healthcare, Piscataway, NJ, USA).

### 2.12. Statistical Analysis

All statistical analyses were performed in the RStudio environment (version 2026.07.1-147; Posit Software, PBC, 2026) using R version 4.5.1 (R Core Team, 2025). Data are presented as mean ± standard deviation (SD) unless otherwise specified. The effects of pH, KCl concentration, and temperature on relative cleavage activity were evaluated using one-way analysis of variance (one-way ANOVA). Assumptions of normality and homogeneity of variances were assessed using the Shapiro–Wilk test and Levene’s test, respectively. Levene’s test was performed using the *car* package [22]. When significant differences were detected, Tukey’s Honest Significant Difference (HSD) test was used for multiple comparisons. Significant group differences were summarized using a compact letter display implemented in the *multcompView* package [23]. Statistical significance was defined as *p* < 0.05. Linear regression analysis was performed to evaluate relationships between continuous variables, and the goodness of fit was assessed using the coefficient of determination (R^2^).

Kinetic parameters were estimated by fitting the Michaelis–Menten equation to initial-rate data using nonlinear least-squares regression implemented in the *drc* package [24].

Data visualization was performed using the *ggplot2* package [25], and figures were assembled using the *patchwork* package [26]. Bar plots show mean ± SD, and regression analyses are presented with fitted regression lines.

## 3. Results

### 3.1. Production of Recombinant SaUNG and Functional Characterization of Catalytic Mutants

The *S. aureus ung* gene was cloned into pET-11a, and the H180A and D59A/H180A mutants were generated by site-directed mutagenesis. Recombinant wild-type and mutant SaUNG proteins were expressed in *E. coli* Rosetta 2 (DE3) and purified to near homogeneity. Protein purity was confirmed by SDS–PAGE (Supplementary Figure S1).

The currently accepted mechanism of UNG catalysis, derived from biochemical, structural, and computational studies of the *E. coli* and human enzymes, involves two conserved active-site residues [27,28]. An aspartate side chain (Asp145 in hUNG2, Asp64 in EcUNG) activates a water molecule for nucleophilic attack at C1’ of the damaged nucleotide. At the same time, a neutral histidine imidazole moiety (His268 in hUNG2, His187 in EcUNG) forms a strong hydrogen bond to O2 of the uracil base, stabilizing the anionic reaction intermediate (Figure 1a). The homologous residues in SaUNG are Asp59 and His180, respectively. Multiple sequence alignment of SaUNG with structurally characterized Family 1 UNGs confirmed the conservation of the principal active-site elements, including the GQDPY and HPSPLS motifs containing Asp59 and His180, respectively, as well as the uracil-recognition and minor-groove base-flipping elements (Figure S2).

Given the conservation of these residues among members of the UNG family, the corresponding SaUNG residues Asp59 and His180 were selected for site-directed mutagenesis. Recombinant SaUNG H180A and SaUNG D59A/H180A mutant proteins were expressed and purified using the same procedure as described for the wild-type enzyme. The DNA glycosylase activities of the purified mutant proteins were subsequently evaluated and compared with that of wild-type SaUNG (Figure 1b).

Wild-type SaUNG efficiently excised uracil from DNA, resulting in the formation of the expected 9-mer cleavage product in a concentration-dependent manner at enzyme concentrations of 0.25–1 nM. In contrast, the double mutant SaUNG D59A/H180A failed to generate detectable cleavage products even at substantially higher concentrations (20–100 nM), indicating that simultaneous substitution of Asp59 and His180 abolished detectable activity under the experimental conditions employed. The single H180A mutant retained residual activity; however, markedly higher enzyme concentrations were required to achieve substrate cleavage comparable to that observed for wild-type SaUNG. Together, these results demonstrate that His180 is important for efficient SaUNG catalysis, whereas simultaneous substitution of Asp59 and His180 abolishes detectable DNA glycosylase activity under the experimental conditions employed. Because the single D59A mutant was not examined, the individual contribution of Asp59 cannot be directly assessed from the present data.

### 3.2. Effects of Ionic Strength, pH, and Temperature on SaUNG DNA Glycosylase Activity

To optimize the reaction buffer for subsequent biochemical analyses, the dependence of SaUNG activity on ionic strength, pH, and temperature was investigated. The dependence of SaUNG activity on ionic strength, pH, and temperature is presented in Figure 2.

SaUNG activity was strongly influenced by KCl concentration, increasing markedly with increasing ionic strength and reaching maximal levels at 100–150 mM KCl. Further increases in KCl concentration resulted in a progressive decline in activity, with substantial inhibition observed at concentrations of 400 mM and above (Figure 2a). SaUNG exhibited a pronounced pH dependence, displaying minimal activity under acidic conditions (pH 4.0–6.0). Enzymatic activity increased progressively toward alkaline pH values and peaked at pH 8.0–8.5, followed by a decrease at pH 9.0 (Figure 2b). Temperature profiling revealed that SaUNG remained active over a broad temperature range. The highest activity was observed at 37–45 °C, whereas lower activity was detected at 4 °C and 23 °C and progressively declined at temperatures above 45 °C (Figure 2c). Based on these findings, SaUNG displayed maximal activity at pH 8.0–8.5, 37–45 °C, and 100–150 mM KCl.

### 3.3. Activity of Recombinant SaUNG

To assess the substrate preference of SaUNG, uracil excision activity was evaluated using DNA substrates containing uracil paired with either guanine or adenine, together with a single-stranded uracil-containing oligonucleotide (ssU) (Figure 3).

SaUNG efficiently processed all substrates tested, generating the expected 9-mer cleavage product. Under the experimental conditions employed, uracil excision from the single-stranded substrate appeared more efficient than from either duplex substrate, whereas U:G and U:A were cleaved with comparable efficiencies. The ability of SaUNG to process both U:G and U:A substrates indicates that the enzyme can remove uracil arising from either cytosine deamination or dUTP misincorporation. The higher activity observed toward single-stranded DNA is consistent with previous observations reported for human UNG [29].

DNA glycosylases can be classified as either monofunctional or bifunctional enzymes. Monofunctional glycosylases catalyze only the excision of damaged bases, thereby generating an AP site, whereas bifunctional glycosylases possess an additional AP lyase activity that cleaves the DNA backbone at the resulting AP site [30]. To determine the functional type of SaUNG, the ability of the enzyme to cleave the DNA backbone following uracil excision was evaluated (Figure 4).

In the absence of alkaline treatment, SaUNG did not generate detectable cleavage products under the experimental conditions employed, whereas alkaline hydrolysis of the reaction products resulted in the appearance of the expected 9-mer fragment. Similar behavior was observed for the monofunctional control enzyme EcUNG. These findings indicate that SaUNG lacks intrinsic AP lyase activity and functions as a monofunctional uracil-DNA glycosylase that excises uracil to generate an AP site requiring subsequent processing by downstream BER enzymes. This behavior closely resembles that of the canonical Family 1 uracil-DNA glycosylase EcUNG [31] and is consistent with the established mechanism of UNG enzymes, which initiate base excision repair by generating an AP site subsequently processed by downstream repair factors.

UGI, a bacteriophage-encoded protein inhibitor, is known to specifically target members of the UNG family through the formation of a tight protein–protein complex that occludes the DNA-binding surface and prevents substrate access to the active site [11]. To investigate the sensitivity of SaUNG to inhibition by the bacteriophage-derived uracil-DNA glycosylase inhibitor UGI, its activity was examined in the presence of the inhibitor. EcUNG, a well-characterized UGI-sensitive uracil-DNA glycosylase [32], was included as a positive control. Prior to the assay, the enzymes were preincubated with UGI to facilitate the formation of enzyme–inhibitor complexes (Figure 5).

Both SaUNG and EcUNG efficiently converted the uracil-containing substrate into the expected cleavage product in the absence of UGI. In contrast, preincubation with UGI resulted in complete inhibition of both SaUNG and EcUNG, with no detectable cleavage products observed under the experimental conditions employed. These results demonstrate that SaUNG is susceptible to UGI inhibition under the tested conditions. This finding is consistent with previous structural and biochemical studies demonstrating that susceptibility to UGI is a characteristic feature of Family 1 uracil-DNA glycosylases [32,33,34] and further supports the assignment of SaUNG to this enzyme family.

To quantitatively compare the catalytic properties of EcUNG and SaUNG, steady-state kinetic analyses were performed using U:G and U:A duplex substrates as well as single-stranded uracil-containing DNA (ssU). Initial reaction rates were measured in the standard reaction buffer (20 mM Tris–HCl (pH 8.0), 10 mM KCl, 0.5 mM EDTA, 0.1 mg/mL BSA, 1 mM DTT) under optimized assay conditions. Apparent kinetic parameters were obtained by nonlinear regression using the Michaelis–Menten equation from two independent experiments and are presented as mean ± SD (Table 2).

As shown in Table 2, both EcUNG and SaUNG efficiently processed all tested substrates but differed in their kinetic properties. EcUNG exhibited slightly higher catalytic efficiency toward the U:G substrate, whereas SaUNG was more efficient toward the U:A substrate. The largest difference was observed for the ssU substrate, for which SaUNG exhibited a lower apparent K_m_ and substantially higher *k_cat_* than EcUNG, resulting in higher catalytic efficiency. Overall, EcUNG displayed the highest catalytic efficiency toward the U:G substrate, whereas SaUNG exhibited the highest catalytic efficiency toward ssU, followed by the U:G and U:A duplex substrates.

### 3.4. Modeling of SaUNG Structure

The structure of SaUNG was solved for the free wild-type protein (Protein Data Bank ID: 3WDF) and for complexes with several proteinaceous inhibitors of viral and bacterial origin (3WDG, 8AIM, 8AIN) [9,13]. However, the structure of SaUNG–DNA complexes and the structural consequences of mutations in key catalytic residues have not yet been characterized experimentally, limiting the ability to compare SaUNG with better-studied enzymes such as hUNG and EcUNG. To gain insight into the mechanism of SaUNG and the effects of the D59A and H180A mutations, we constructed models of wild-type SaUNG, SaUNG H180A and SaUNG D59A/H180A, either free or DNA-bound. Five AlphaFold 3 models were generated for each protein species and each complex. Since AlphaFold 3 does not support U in DNA, we replaced it with an AP site and used the DNA sequence from the structure of human UNG bound to the reaction product, i.e., an AP site-containing duplex and a uracil base (1EMJ [19]). This allowed the models to recapitulate the highly bent DNA conformation characteristic of DNA glycosylase complexes.

Both wild-type and mutant models demonstrated high pLDDT confidence values except for the last three C-terminal residues and a small ^76^PNAK^79^ turn in the middle of the free protein (Supplementary Figure S3) and coincided almost exactly both within each group of models (Cα RMSD 0.109–0.396 Å for the free proteins and 0.179–0.726 Å for the DNA-bound proteins) and between groups (Cα RMSD 0.090–0.480 Å for the free proteins and 0.156–0.735 Å for the DNA-bound proteins). The models of free SaUNG overlapped well with the experimental structure (Cα RMSD 0.313–0.479 Å for wild-type, 0.356–0.464 Å for H180A, and 0.315–0.371 Å for D59A/H180A), and those of DNA-bound SaUNG, with the crystal structure of DNA-bound hUNG (Cα RMSD 1.312–1.331 Å for wild-type, 1.304–1.339 Å for H180A, and 1.318–1.340 Å for D59A/H180A; Figure 6a–c). However, DNA binding and the mutations introduced some important changes, evident from the analysis of the main-chain traces, hydrogen-bond networks, and p*K*_a_ values of titratable groups in the models.

Although DNA binding was not accompanied with gross structural changes in the SaUNG molecule, two conformational adjustments seemed to be functionally relevant. First, the main chain between Leu115 and Asn116 rotated, drawing the Asn116 side chain deeper into the uracil-binding pocket of the enzyme, apparently to make better contacts with the excised base (Figure 6d). Second, the closure of the protein on DNA moved a long loop (Ser73–Ser83) bearing the ^76^PNAK^79^ turn and the “proline pinch” motif (Figure 6e), which, based on the structure of hUNG, is used by the enzyme to compress the DNA backbone and flip uracil out of the helix [35]. This movement also assisted the insertion of the plugging Leu184 into DNA and caused a local rearrangement in the vicinity of Asp59, uncoupling the side chains of Gln58 and Asn127. As a result, DNA binding increased the calculated p*K*_a_ of the catalytic Asp59 from 0.8 ± 0.1 to 3.9 ± 0.5 (mean ± SD over all five models), priming it for water activation, and decreased the p*K*_a_ of His180 (Nδ1) from 2.6 ± 0.1 to 1.0 ± 0.3, which would facilitate the donation of a hydrogen bond by the Nε2 atom to the uracil base important for catalysis [36,37]. The anomalously low p*K*_a_ of the homologous His residue was shown by NMR for EcUNG [36] and is most likely due to Nδ1 involved in a strong hydrogen bond with the main chain amide of the Ser residue in the absolutely conserved HPSP turn (Supplementary Figure S2), which we also observe in our models (Figure 6e). In the SaUNG H180A mutant, p*K*_a_ of Asp59 slightly increased (to 1.4 ± 0.2) in the free protein but not in the complex (4.0 ± 0.3), suggesting that even in the absence of the catalytic His180, Asp59 can still activate the water molecule, consistent with the residual activity of the single mutant, and that the decrease in the reaction rate is likely due to the impeded departure of the unprotonated uracil.

### 3.5. Role of SaUNG in Mutation Avoidance In Vivo

To assess whether SaUNG contributes to the prevention of spontaneous mutagenesis *in vivo*, mutation rates were determined in the *E. coli* MS1022 (*ung^−^ mug^−^*) strain expressing EcUNG, SaUNG, or the catalytically impaired SaUNG D59A/H180A variant. The results are shown in Figure 7.

The *E. coli* MS1022 (*ung^−^ mug^−^*) strain carrying the empty pBSK vector exhibited the highest spontaneous mutation rate. Expression of either EcUNG or SaUNG significantly reduced spontaneous mutagenesis relative to the vector control. No statistically significant difference was observed between strains expressing EcUNG and wild-type SaUNG, indicating that SaUNG efficiently suppresses spontaneous mutations *in vivo*. In contrast, expression of the SaUNG D59A/H180A mutant resulted in a significantly higher mutation rate than that observed for wild-type SaUNG, although the mutation frequency remained lower than that of the vector control.

These findings indicate that SaUNG can efficiently compensate for the deficiency in endogenous uracil-DNA glycosylase activity in *E. coli* and demonstrate that the enzyme is capable of suppressing uracil-associated spontaneous mutagenesis *in vivo*. Expression of the catalytically impaired D59A/H180A mutant failed to restore mutation avoidance to the same extent as wild-type SaUNG, demonstrating that efficient suppression of spontaneous mutagenesis depends on the catalytic activity of the enzyme. The significantly higher mutation rate observed for the mutant compared with wild-type SaUNG further supports a relationship between SaUNG catalytic activity and mutation prevention *in vivo*.

### 3.6. Reconstitution of Uracil Repair in S. aureus

To assess the ability of SaUNG to function in combination with other *S. aureus* BER enzymes, we reconstituted uracil repair using purified proteins, including *S. aureus* endonuclease IV (SaNfo), DNA polymerase X (SaPolX), and β-clamp protein (SaDnaN), whose purity was verified by SDS–PAGE (Supplementary Figure S1). SaNfo is the main *S. aureus* AP endonuclease, and has been reported to be stimulated by SaDnaN [15,38]. SaPolX belongs to the X family of DNA polymerases, which includes enzymes participating in BER and non-homologous end joining, such as human DNA polymerases β, λ, and μ and terminal deoxynucleotidyl transferase [39,40]. Phage T4 DNA ligase was used to reveal ligatable DNA nicks after the polymerase step, as commonly employed in BER reconstitution assays [41,42,43,44]. When presented with a 23 bp U:A duplex, SaUNG efficiently formed an alkali-labile AP site, which was then cleaved by SaNfo to produce a slower-migrating species lacking the negatively charged 3′-terminal phosphate (Figure 8, lanes 1–3). SaPolX then incorporated a dTMP residue and formed a ligatable nick (Figure 8, lanes 4–5). Increasing the amount of SaNfo allowed more complete ligation but was accompanied by nonspecific exonuclease digestion (Supplementary Figure S4). Post-treatment of the reaction products with EcUNG produced no additional cleavage, confirming that U was fully replaced with T in the substrate duplex (Figure 8, lanes 6, 8). SaDnaN did not significantly enhance repair in the complete system (Figure 8, lanes 7–8). Hence, SaUNG is fully proficient in initiating a BER round that can be successfully continued by other *S. aureus* repair proteins.

## 4. Discussion

Uracil-DNA glycosylases catalyze the first and essential step of the base excision repair pathway by recognizing and excising uracil from DNA generated either by cytosine deamination or by dUTP misincorporation during replication [30]. Although UNG enzymes have been extensively characterized in *E. coli* and humans [31,45], comparatively little is known about the biochemical properties and catalytic mechanism of the staphylococcal enzyme. Genome sequencing identified a single *ung* gene encoding a predicted Family 1 uracil-DNA glycosylase [46]. At present, the best-studied aspect of SaUNG is its interactions with inhibitors of Family 1 uracil–DNA glycosylases from *S. aureus* itself, several closely related bacterial genera (*Macrococcus*, *Salinicoccus*, *Jeotgalicoccus*), and several bacteriophages infecting *Bacillus* hosts (PBS1/2, *ϕ*29, vB_BpuM-BpSp, PZA, VMY22) [8,9,13,14]. Regarding catalytic activity, only the ability of the enzyme to degrade uracil-containing DNA has been demonstrated [9,13], without further mechanistic characterization. The structure of free SaUNG has been solved as part of efforts to characterize its binding to these inhibitors [9], but neither experimental nor modeling information on SaUNG–DNA interactions is available. The present study extends this previous work by providing detailed kinetic characterization of SaUNG, mutational analysis of its catalytic residues, and *in vitro* reconstitution of the *S. aureus* BER pathway.

Asp59 is located within the conserved catalytic GQDPY motif [31], which is known to participate in the activation and positioning of the nucleophilic water molecule required for cleavage of the N-glycosidic bond. Structural and biochemical studies of bacterial and eukaryotic Family 1 UNGs have demonstrated that substitution of the corresponding conserved aspartate severely impairs catalytic activity, supporting its central role in uracil excision [35,47]. His180 belongs to the highly conserved HPSPLS motif [31], which contributes to DNA binding, nucleotide flipping, and stabilization of the enzyme–substrate complex. In the present study, the H180A variant retained residual activity, although substantially higher enzyme concentrations were required to achieve substrate cleavage compared with wild-type SaUNG, indicating that His180 is important for efficient SaUNG catalysis. Simultaneous substitution of Asp59 and His180 in the D59A/H180A mutant abolished detectable DNA glycosylase activity under the experimental conditions employed. However, because the single D59A mutant was not examined, the individual contribution of Asp59 cannot be directly assessed from the present data. Although the catalytic role of the corresponding conserved aspartate is well established in other Family 1 UNGs, characterization of the single SaUNG D59A mutant will be required to experimentally distinguish its individual contribution from the combined effect observed for the D59A/H180A mutant.

Biochemical characterization further demonstrated that SaUNG exhibits maximal activity at pH 8.0–8.5, 37–45 °C, and 100–150 mM KCl. These catalytic properties are generally consistent with those reported for human UNG and other Family 1 uracil-DNA glycosylases, including enzymes from *Plasmodium falciparum* and *Trypanosoma cruzi*, which also exhibit high or maximal activity under slightly alkaline conditions [29,48,49].

In the absence of an experimental SaUNG–DNA structure, the AlphaFold 3 models may provide some structural insight into the catalytic mechanism of SaUNG. Overall, the enzyme appears to be a typical Family 1 uracil–DNA glycosylase, a group to which well-characterized UNGs from humans and *E. coli* belong. The positions of the catalytic Asp–His dyad, the uracil-recognizing Asn and Tyr residues within the active site, and the DNA helix-invading Leu side chain are conserved. Hydrogen-bonding and steric restrictions imposed by Asn116 and Tyr61 would prohibit the excision of normal Thy and Cyt and any other damaged base except for uracil. The models also recapitulated the proline pinch movement and the backbone adjustment in the uracil-binding pocket upon DNA binding observed in human UNG [19,35]. Interestingly, the loop near the proline pinch motif had the lowest pLDDT score among the internal regions of the protein, suggesting that this region may be flexible. The models also allowed us, while acknowledging the limitations of protein structure prediction, to propose a possible explanation for the partially retained activity in the SaUNG H180A mutant discussed above: the p*K*_a_ of Asp59 in the DNA-bound wild-type and H180A enzyme remained similar, which would presumably allow Asp59 to activate the water molecule for nucleophilic attack at C1′ even in the absence of His180. Further mechanistic details should become available once the actual structure of the SaUNG–DNA complex is solved and the behavior of the catalytic residues is probed by direct methods such as nuclear magnetic resonance or electron paramagnetic resonance spectroscopy [50,51,52].

SaUNG efficiently excised uracil from U:G and U:A duplexes as well as from single-stranded DNA, consistent with its role in repairing uracil arising from cytosine deamination and dUTP misincorporation. Kinetic analysis revealed the highest catalytic efficiency toward ssU, followed by U:G and U:A, whereas EcUNG showed the highest efficiency toward U:G. The substrate preference of SaUNG (ssU > U:G > U:A) is consistent with that reported for *T. cruzi* and human UNGs [48,53]. Furthermore, SaUNG functions as a monofunctional DNA glycosylase lacking detectable AP-lyase activity, with DNA strand cleavage occurring only after alkaline treatment of the abasic intermediate, consistent with the catalytic mechanism of Family 1 uracil-DNA glycosylases [31]. Complete inhibition by UGI, together with the indistinguishable sensitivity of SaUNG and EcUNG, further supports conservation of the molecular determinants required for inhibitor recognition. Collectively, the broad substrate specificity, monofunctional catalytic mechanism, and conserved susceptibility to UGI support the classification of SaUNG as a canonical Family 1 uracil-DNA glycosylase that employs the conserved catalytic strategy characteristic of this enzyme family. Although EcUNG and SaUNG differed in their substrate-dependent kinetic properties, both enzymes efficiently processed the tested uracil-containing substrates, indicating conservation of the overall catalytic function of UNG despite species-specific kinetic differences.

SaUNG efficiently complemented the mutator phenotype of an *E. coli* (*ung^–^ mug^–^*) mutant, restoring spontaneous mutation frequencies to levels comparable with those achieved by EcUNG. In contrast, the catalytically impaired D59A/H180A mutant failed to suppress spontaneous mutagenesis efficiently, supporting a link between uracil excision activity and genome stability *in vivo*. These findings are consistent with the established role of UNG-dependent base excision repair in preventing spontaneous transition mutations arising from uracil in DNA [7].

Reconstitution of the *S. aureus* uracil repair pathway using purified SaUNG, SaNfo, SaPolX, and SaDnaN demonstrates that SaUNG efficiently initiates base excision repair and functions in concert with downstream repair proteins. Although SaDnaN did not measurably stimulate repair under the experimental conditions used, its physiological contribution may require additional replication factors or more complex DNA substrates. This work represents the first biochemical reconstitution of uracil-initiated BER using purified *S. aureus* proteins and establishes a foundation for future mechanistic studies of genome maintenance in this pathogen.

Beyond defining the biochemical properties of a single DNA glycosylase, our findings have broader implications for understanding genome maintenance in *S. aureus*. During infection, *S. aureus* is continuously challenged by reactive oxygen and nitrogen species generated by host phagocytes, together with endogenous metabolic stress, creating a persistent requirement for efficient DNA repair to maintain genome integrity [2,54]. Although oxidative lesions are primarily repaired by other BER glycosylases, spontaneous cytosine deamination and replication-associated uracil incorporation generate uracil lesions that require efficient removal by UNG. Recent studies have highlighted the importance of DNA repair pathways for *S. aureus* survival during infection, contributing to genome maintenance, adaptation to host-imposed stress, and the emergence of antibiotic resistance [55,56]. In this context, the efficient catalytic activity of SaUNG, its *in vivo* complementation, and the successful reconstitution of BER using purified *S. aureus* proteins support its functional role in the staphylococcal DNA repair network. Together with growing interest in bacterial DNA repair pathways as potential targets for antimicrobial intervention [56,57,58], our findings provide a biochemical framework for future studies aimed at understanding how uracil repair contributes to staphylococcal persistence and whether interference with BER may sensitize *S. aureus* to host immune defenses or conventional antibiotics.

## 5. Conclusions

This study confirms that SaUNG is a canonical Family 1 uracil-DNA glycosylase. SaUNG efficiently excised uracil from both double- and single-stranded DNA and lacked detectable AP-lyase activity, consistent with its function as a monofunctional DNA glycosylase. Steady-state kinetic analysis revealed the highest catalytic efficiency toward ssU, followed by U:G and U:A duplex substrates. Mutational analysis showed that the H180A variant retained residual activity, whereas simultaneous substitution of Asp59 and His180 abolished detectable DNA glycosylase activity under the experimental conditions employed. Complete inhibition by UGI was consistent with the characteristic sensitivity of Family 1 UNGs to this inhibitor. In addition, SaUNG reduced the mutation rate in an *E. coli* (*ung^−^ mug^−^*) mutant and efficiently initiated uracil repair in a reconstituted *S. aureus* base excision repair pathway. Together, these findings provide further information on the biochemical properties, catalytic mechanism, and biological function of SaUNG and establish a foundation for further studies of uracil repair and base excision repair in *S. aureus*.

## Figures and Tables

**Figure 1:**
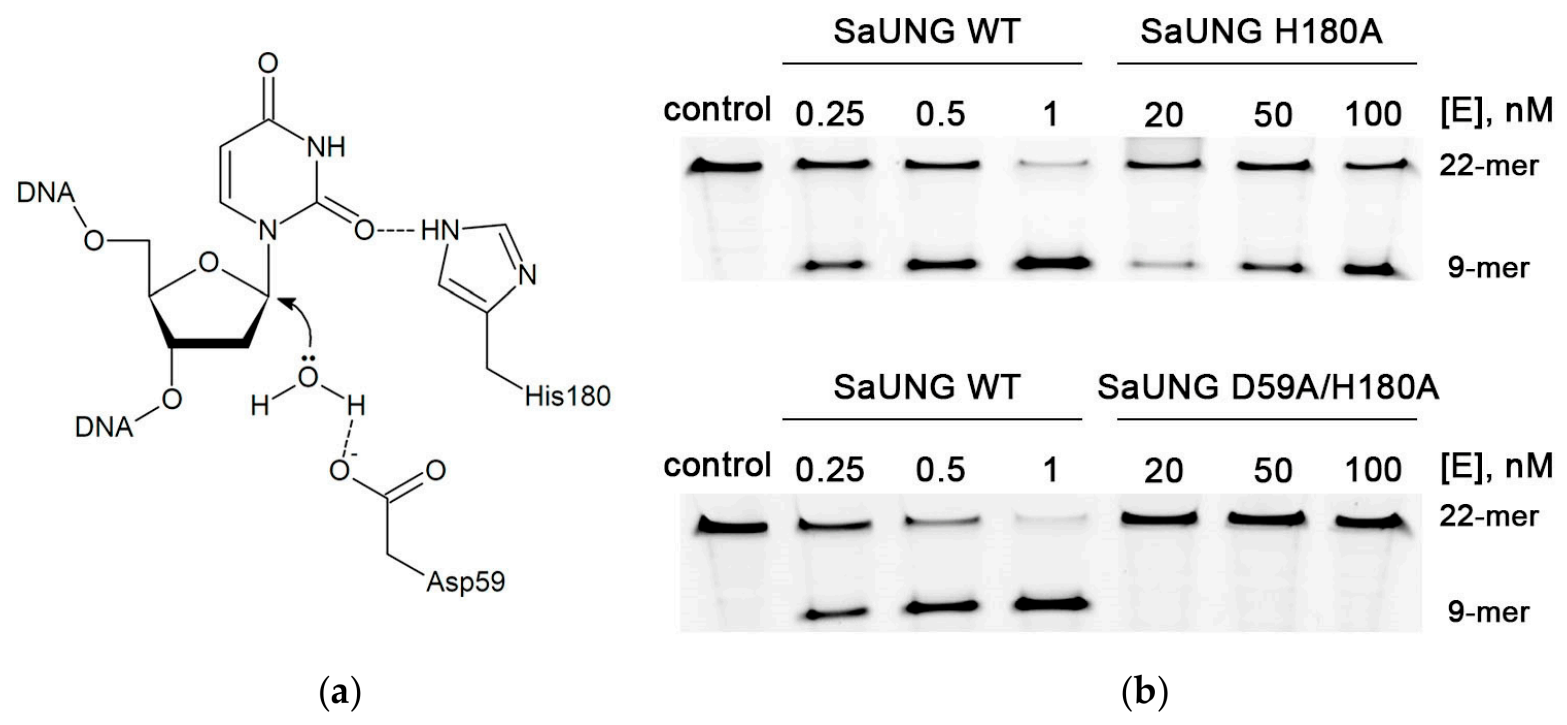
Mutational analysis of conserved active-site residues in SaUNG. (**a**) Schematic representation of the proposed catalytic mechanism highlighting the roles of Asp59 and His180 in uracil excision. (**b**) Comparison of the DNA glycosylase activities of wild-type SaUNG and the H180A and D59A/H180A mutants.

**Figure 2:**
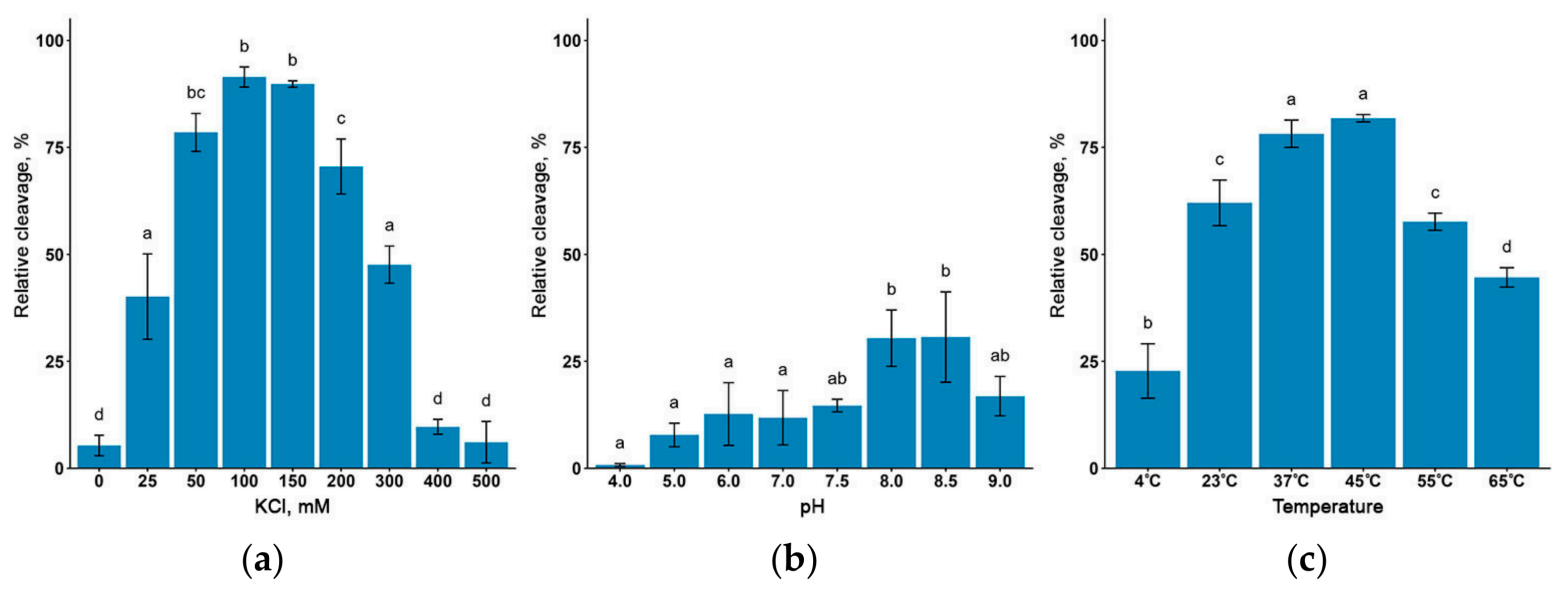
Effects of KCl concentration, pH, and temperature on SaUNG activity. (**a**) Effect of KCl concentration (5–500 mM). Reactions contained 0.25 nM SaUNG and were incubated at 37 °C for 3 min. (**b**) Effect of pH. Sodium acetate buffer was used at pH 4.0 and 5.0, sodium phosphate buffer at pH 6.0, Tris–HCl buffer at pH 7.0, 7.5, 8.0, and 8.5, and glycine–NaOH buffer at pH 9.0. Reactions contained 2 nM SaUNG and were incubated at 37 °C for 5 min. (**c**) Effect of temperature (4–65 °C). Reactions contained 1 nM SaUNG and were incubated for 1 min. Data are presented as mean ± SD of three independent experiments. Different letters indicate statistically significant differences among tested conditions (*p* < 0.05, one-way ANOVA followed by Tukey’s HSD test).

**Figure 3:**
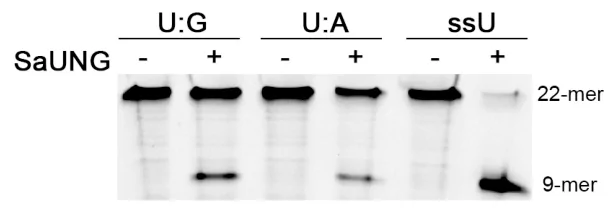
Substrate specificity of SaUNG toward uracil-containing DNA substrates.

**Figure 4:**
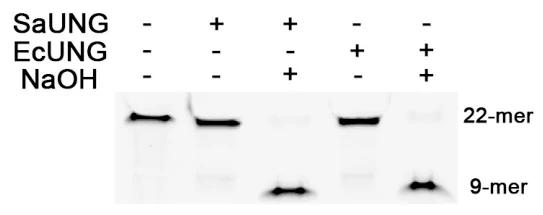
Assessment of the AP lyase activity of SaUNG.

**Figure 5:**
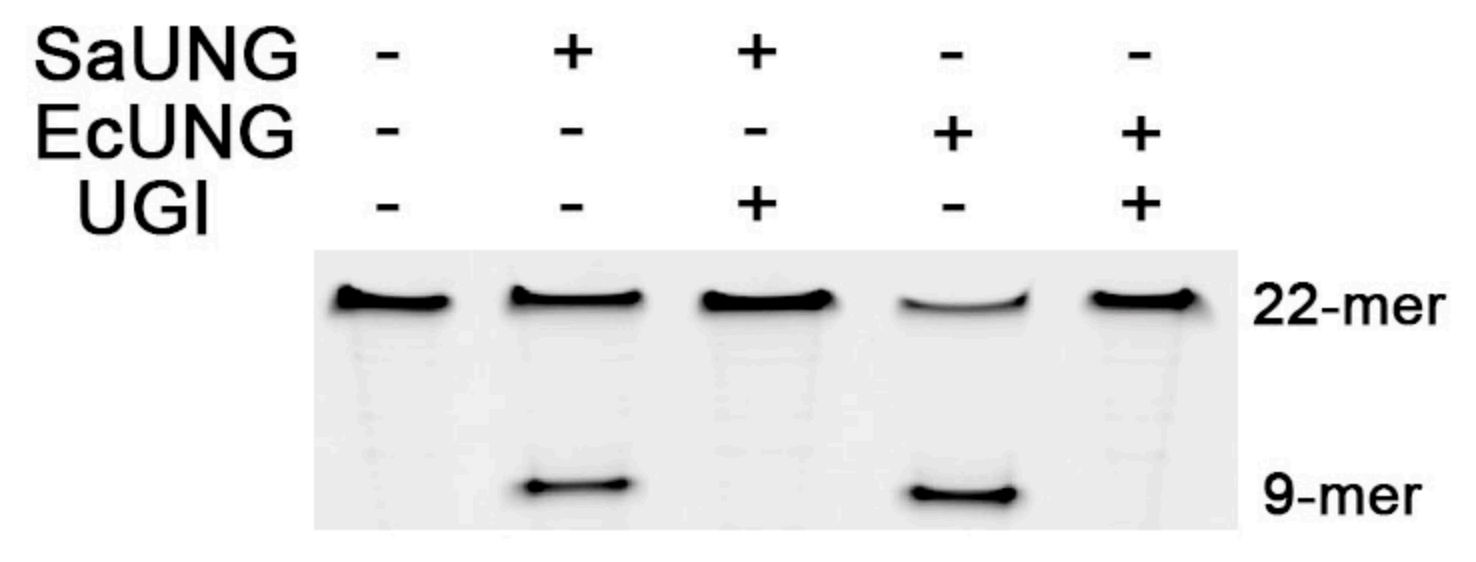
Inhibition of SaUNG activity by uracil-DNA glycosylase inhibitor (UGI).

**Figure 6:**
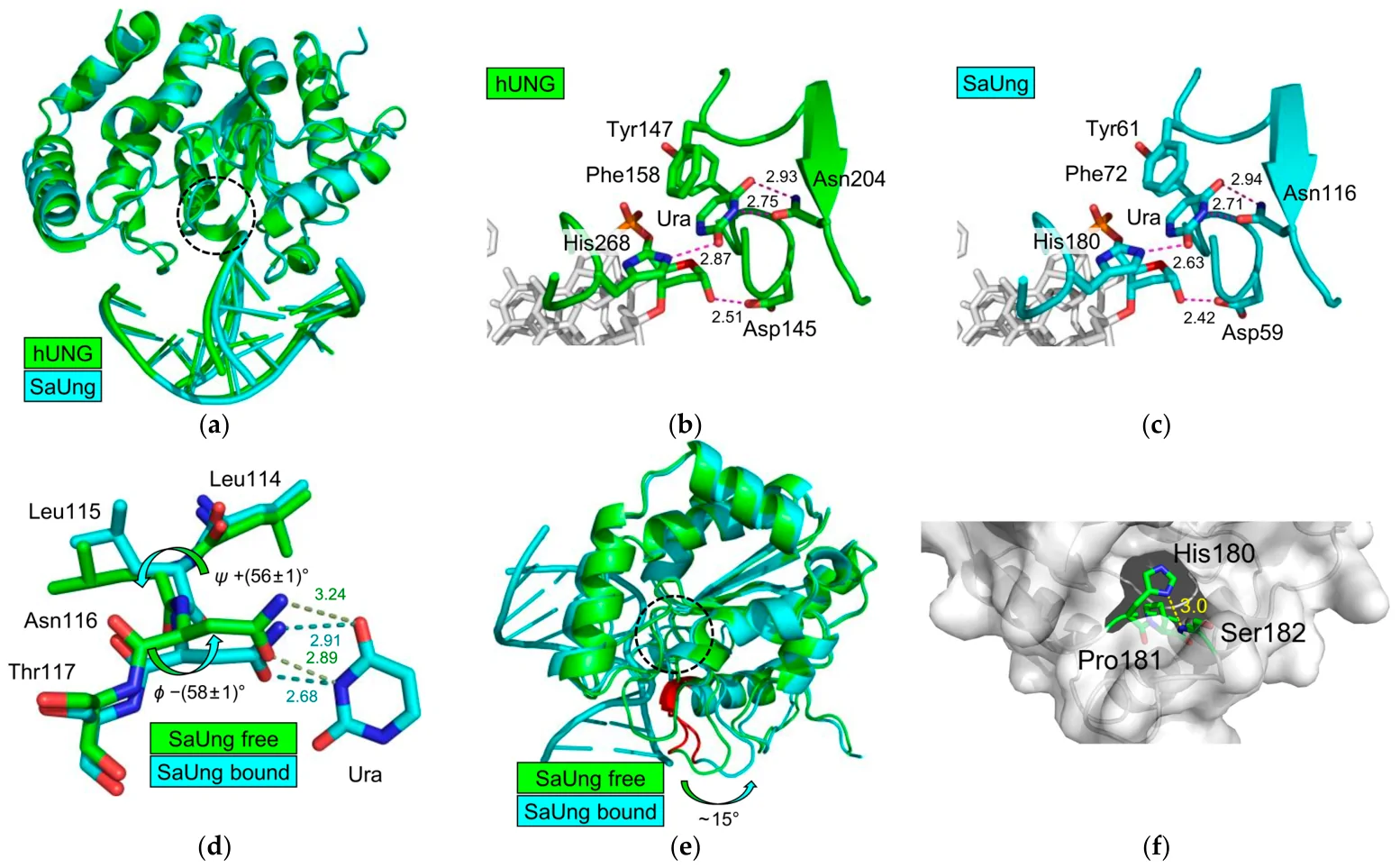
Structural model of SaUNG. (**a**) Overlay of the crystal structure of DNA-bound hUNG (green, PDB ID: 1EMJ [19] and the best-scoring AlphaFold 3 model of DNA-bound SaUNG (cyan). (**b**) Contacts formed with the excised uracil base and the nascent AP site in the active site of hUNG. (**c**) Contacts formed with the excised uracil base in the active site of SaUNG. (**d**) Backbone rotation between Leu115 and Asn116 in SaUNG upon DNA binding. (**e**) Overlay of the best-scoring models of free SaUNG (green) and DNA-bound SaUNG (cyan). The proline pinch motif is shown in red. (**f**), structure around His180 showing a hydrogen bond between its Nδ1 and the main chain amide of Ser182. Ura, uracil. In (**b**–**d**,**f**), oxygen atoms are colored red, nitrogen atoms, blue; hydrogen-bond lengths (in Å) are indicated. In (**a**,**e**), the active site of the enzyme is encircled. In (**e**), the angle between the planes defined by the Cα atoms of Ser73, Ser83, and Pro76 constituting, respectively, two base vertices and the tip of the loop moved by DNA binding is indicated.

**Figure 7:**
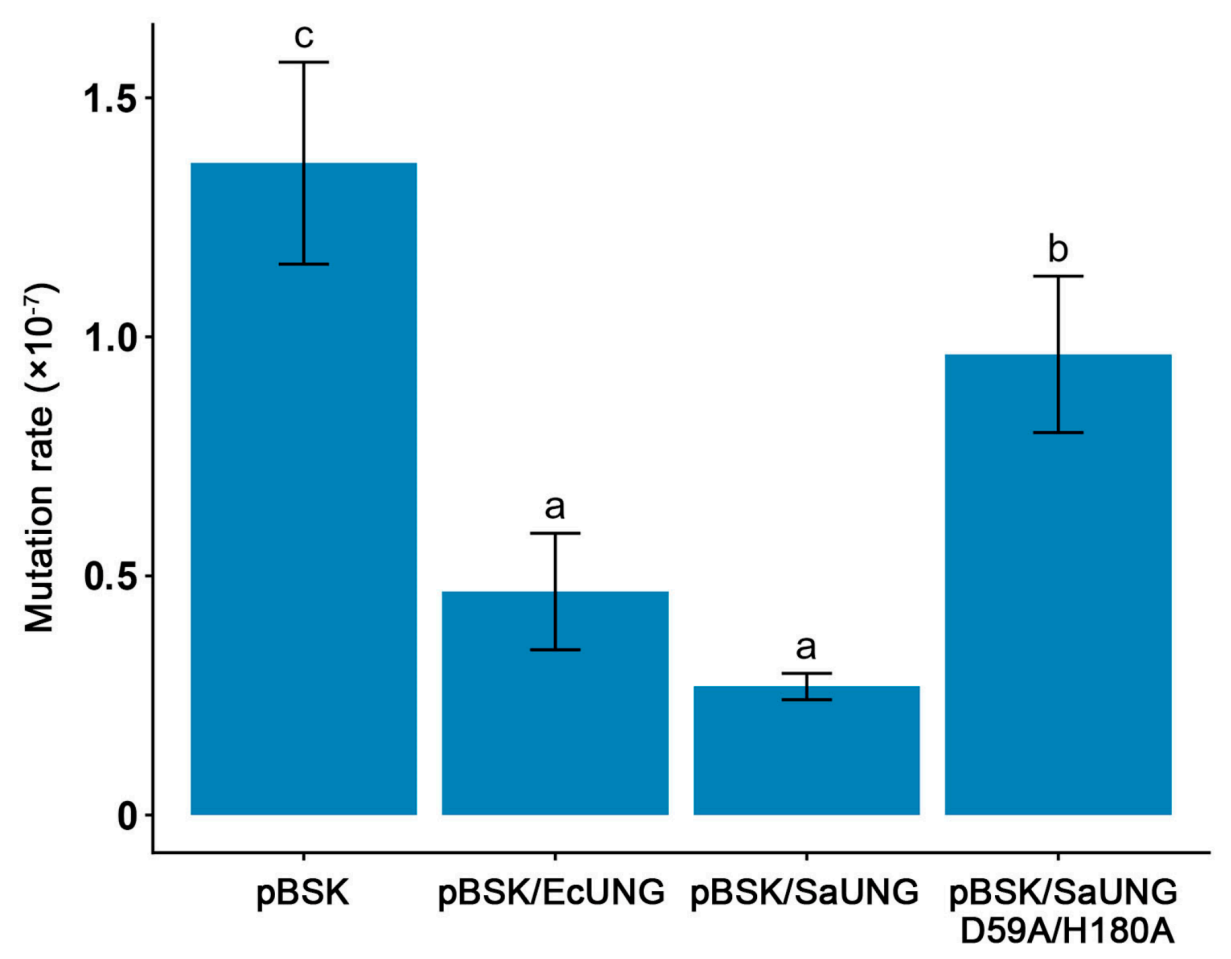
Effect of SaUNG expression on spontaneous mutation rates in *E. coli* MS1022 (*ung^−^ mug^−^*). Data are presented as mean ± SD of independent experiments. Different letters indicate statistically significant differences among groups (*p* < 0.05, one-way ANOVA followed by Tukey’s HSD test).

**Figure 8:**
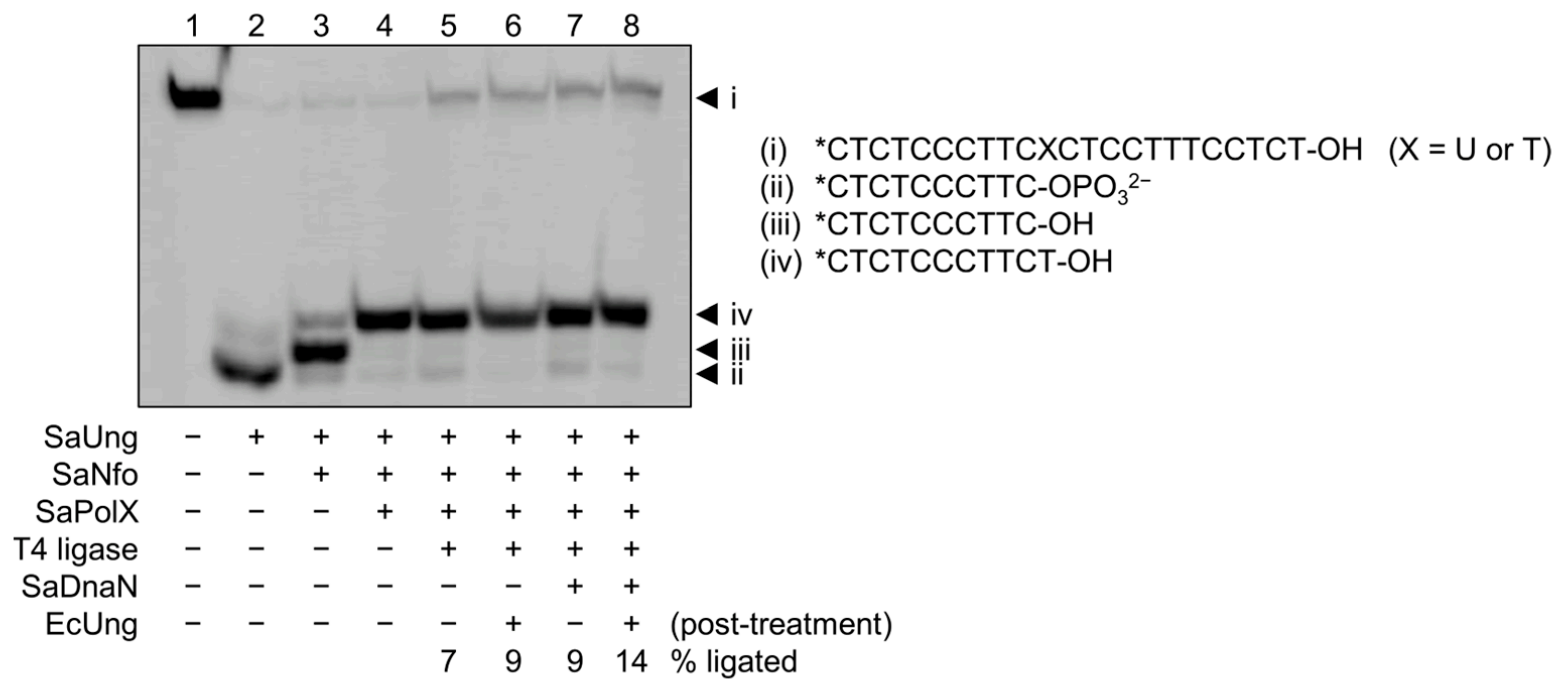
Reconstitution of *S. aureus* uracil BER from purified proteins. Asterisks denote the 5′-terminal fluorescent label.

**Table 1:** Oligonucleotides used in this study.

Oligonucleotide	Sequence (5′ → 3′)
Gene amplification	
NdeI-SaUNG fw	GGAATTCCATATGGAATGGTCGCAAATTTTTC
BamHI-SaUNG rv	CGCGGATCCCTACGCCTCACTTTCACACC
BamHI-rbsSaUNG fw	CGCGGATCCAAGGAGATATAATGGAATGGTCCAAATTTTTC
NotI-SaUNG rv	ATAGTTTAGCGGCCGCCTACGCCTCACTTTCAC
Site-directed mutagenesis	
SaUNG_D59A_fw	GTTATATTAGGACAAGCCCCGTATCATGGTCC
SaUNG_D59A_rv	GGACCATGATACGGGGCTTGTCCTAATATAAC
SaUNG_H180A_fw	GTATTATAAAATCAGTGGCTCCTAGTCCACTGTCTGC
SaUNG_H180A_rv	GCAGACAGTGGACTAGGAGCCACTGATTTTATAATAC
DNA repair activity	
U-22-TAMRA	[TAMRA]CACTTCGGAUTGTGACTGATCC
22-Compl-A	GGATCAGTCACAATCCGAAGTG
22-Compl-G	GGATCAGTCACAGTCCGAAGTG
U-75-TAMRA	[TAMRA]CATTACAGCGCCTGCAAGCTCAATTAATCGTAUTGCTACACTGGAGTACCGGAGCGTCGTCATGACAACGAATGG
U-75	CATTACAGCGCCTGCAAGCTCAATTAATCGTAUTGCTACACTGGAGTACCGGAGCGTCGTCATGACAACGAATGG
75-compl-A	CCATTCGTTGTCATGACGACGCTCCGGTACTCCAGTGTAGCAATACGATTAATTGAGCTTGCAGGCGCTGTAATG
75-compl-G	CCATTCGTTGTCATGACGACGCTCCGGTACTCCAGTGTAGCAGTACGATTAATTGAGCTTGCAGGCGCTGTAATG
U-23-Fluo	[Fluo]CTCTCCCTTCUCTCCTTTCCTCT
23-Compl-A	AGAGGAAAGGAGAGAAGGGAGAG

[TAMRA], 5(6)-carboxytetramethylrhodamine; [Fluo], 5(6)-carboxyfluorescein.

**Table 2:** Comparison of the steady-state kinetic parameters of EcUNG and SaUNG for U:G-, U:A-containing duplex DNA and single-stranded uracil-containing DNA (ssU).

Substrate	Enzyme	K_m_, nM	*k_cat_,* min^−1^	*k_cat_*/K_m_, M^−1^·min^−1^
U:G	EcUNG	64.88 ± 4.98	28.46 ± 0.09	(4.40 ± 0.32) × 10^8^
	SaUNG	83.86 ± 2.85	31.74 ± 1.36	(3.79 ± 0.29) × 10^8^
U:A	EcUNG	64.95 ± 10.63	11.63 ± 0.45	(1.81 ± 0.23) × 10^8^
	SaUNG	51.37 ± 3.70	15.04 ± 0.20	(2.93 ± 0.17) × 10^8^
ssU	EcUNG	447.20 ± 121.36	55.38 ± 5.24	(1.27 ± 0.23) × 10^8^
	SaUNG	203.10 ± 11.11	282.45 ± 8.86	(1.39 ± 0.32) × 10^9^

## Data Availability

The original contributions presented in this study are included in the article/Supplementary Materials. Further inquiries can be directed to the corresponding author(s).

## Supplementary Materials

The following supporting information can be downloaded at https://www.mdpi.com/article/10.3390/pathogens15101012/s1, Figure S1: SDS–PAGE analysis of recombinant proteins used in this study. Coomassie Blue-stained 12% SDS–polyacrylamide gel (Laemmli system). (A) Recombinant proteins used in biochemical assays. *Lane 1*, protein molecular weight marker (Precision Plus Protein™ All Blue Prestained Protein Standards, Bio-Rad); arrowheads indicate molecular masses (kDa). *Lane 2*, SaUNG; *lane 3*, SaUNG H180A; *lane 4*, SaUNG D59A/H180A. (B) Recombinant proteins used for *in vitro* reconstitution of the *S. aureus* base excision repair pathway. *Lane 1*, protein molecular weight marker (PageRuler™ Prestained Protein Ladder, Thermo Fisher Scientific); *lane 2*, SaNfo; *lane 3*, SaUNG; *lane 4*, SaDnaN; *lane 5*, SaPolX. Figure S2: Alignment of sequences of Family 1 UNG proteins for which crystal structures are available. Conserved motifs are delineated according to [35]: cyan, water-activating loop; red, 4-Pro loop, also known as the proline pinch; magenta, uracil recognition strand; blue, Gly–Ser loop; green, Leu272 loop (hUNG numbering), starting with the HPSP turn. Arrowheads mark the catalytic Asp–His dyad. The represented species are: SaUng, *S. aureus*; EcUng, *E. coli*; VcUng, *Vibrio cholerae*; CbUng, *Coxiella burnetii*; BsUng, *Bacillus subtilis*; MtUng, *Mycobacterium tuberculosis*; DrUng, *Deinococcus radiodurans*; hUNG, *Homo sapiens*; cUNG, *Gadus morhua* (Atlantic cod); lUNG, *Leishmania naiffi*; HSVUNG, herpes simplex virus 1; EBVUNG, Epstein–Barr virus; KSVUNG, Kaposi’s sarcoma-associated herpesvirus; mvUNG, *Acanthamoeba polyphaga* mimivirus. Figure S3: Predicted Local Distance Difference Test (pLDDT) values for AlphaFold 3-generated models of SaUNG. pLDDT is a per-atom confidence estimate on a 0–100 scale, where a higher value indicates higher confidence. A, free wild-type SaUNG; B, free SaUNG H180A; C, free SaUNG D59A/H180A; D, wild-type SaUNG–DNA, the protein part of the complex; E, SaUNG H180A–DNA, the protein part of the complex; F, SaUNG D59A H180A–DNA, the protein part of the complex; G, wild-type SaUNG–DNA, the DNA part of the complex; H, SaUNG H180A–DNA, the DNA part of the complex; I, SaUNG D59A H180A–DNA, the DNA part of the complex. Each dot shows pLDDT for the Cα (protein) or C1′ (DNA) atoms at the respective position in one model; most of the dots overlap due to similar pLDDT values. The dashed lines show the threshold levels of structure prediction confidence; the confidence interpretation is presented in Panel A. The interface predicted template modeling (ipTM) score for all complexes was 0.96, with protein–DNA chain pair ipTMs in the 0.94–0.97 range. The ipTM score ranges from 0 to 1, with values >0.8 indicating a confidently predicted interaction. Figure S4: Reconstitution of the *S. aureus* uracil base excision repair pathway. The assay was performed using purified proteins with an increased amount of SaNfo (150 nM). Asterisks denote the 5′-terminal fluorescent label.

## Abbreviations

The following abbreviations are used in this manuscript:

AP site	apurinic/apyrimidinic site
ATP	adenosine triphosphate
BER	base excision repair
BSA	bovine serum albumin
DTT	dithiothreitol
dTTP	deoxythymidine triphosphate
EDTA	ethylenediaminetetraacetic acid
Fluo	5(6)-carboxyfluorescein
IPTG	isopropyl β-D-1-thiogalactopyranoside
MRSA	methicillin-resistant *Staphylococcus aureus*
PAGE	polyacrylamide gel electrophoresis
OD_600_	optical density at 600 nm
pLDDT	predicted Local Distance Difference Test
RMSD	Root Mean Square Deviation
SAUGI	*S. aureus* uracil-DNA glycosylase inhibitor
SD	standard deviation
ssU	single-stranded uracil-containing DNA substrate
TAMRA	5(6)-carboxytetramethylrhodamine
TBE	Tris–Borate–EDTA
UGI	uracil-DNA glycosylase inhibitor
UNG	uracil-DNA glycosylase
