# Supplementary material for: Functional Characterization of Uracil-DNA Glycosylase from Staphylococcus aureus
Source: Pathogens. 2026 Sep 24;15(10):1012. doi: 10.3390/pathogens15101012 (PMC13708272; doi:10.3390/pathogens15101012)

Supplementary Figures
Figure S1

SDS–PAGE analysis of recombinant proteins used in this study. Coomassie Blue-stained 12% SDS–polyacrylamide gel (Laemmli system). (A) Recombinant proteins used in biochemical assays. *Lane 1*, protein molecular weight marker (Precision Plus Protein™ All Blue Prestained Protein Standards, Bio-Rad); arrowheads indicate molecular masses (kDa). *Lane 2*, SaUNG; *lane 3*, SaUNG H180A; *lane 4*, SaUNG D59A/H180A. (B) Recombinant proteins used for *in vitro* reconstitution of the *S. aureus* base excision repair pathway. *Lane 1*, protein molecular weight marker (PageRuler™ Prestained Protein Ladder, Thermo Fisher Scientific); *lane 2*, SaNfo; *lane 3*, SaUNG; *lane 4*, SaDnaN; *lane 5*, SaPolX.

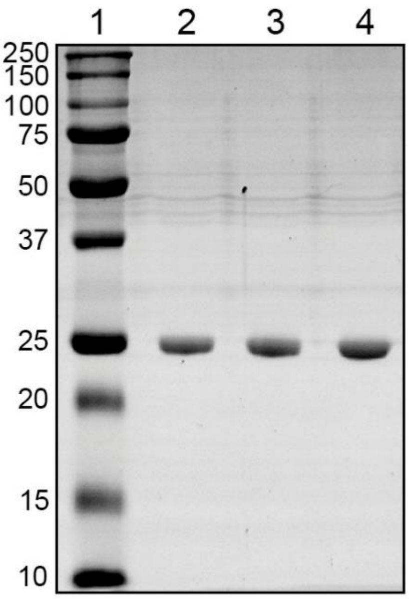

A

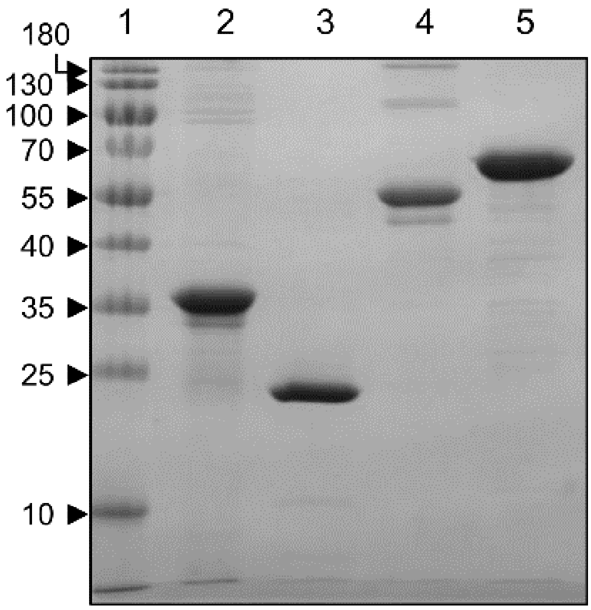

B

Figure S2

Alignment of sequences of Family 1 UNG proteins for which crystal structures are available. Conserved motifs are delineated according to [35]: cyan, water-activating loop; red, 4-Pro loop, also known as the proline pinch; magenta, uracil recognition strand; blue, Gly–Ser loop; green, Leu272 loop (hUNG numbering), starting with the HPSP turn. Arrowheads mark the catalytic Asp–His dyad. The represented species are: SaUng, *S. aureus*; EcUng, *E. coli*; VcUng, *Vibrio cholerae*; CbUng, *Coxiella burnetii*; BsUng, *Bacillus subtilis*; MtUng, *Mycobacterium tuberculosis*; DrUng, *Deinococcus radiodurans*; hUNG, *Homo sapiens*; cUNG, *Gadus morhua* (Atlantic cod); lUNG, *Leishmania naiffi*; HSVUNG, herpes simplex virus 1; EBVUNG, Epstein–Barr virus; KSVUNG, Kaposi's sarcoma-associated herpesvirus; mvUNG, *Acanthamoeba polyphaga* mimivirus.

```
                                                               ▼
SaUng    [14] DFKAMHDFLEKEYST-AIVYPDRENIYQAFDLTPFENIKVVILGQDPYHGP-----NQAHGLAFSVQPNAK-FPPSLRNM   87
EcUng    [18] YFLNTLQTVASERQSGVTIYPPQKDVFNAFRFTELGDVKVVILGQDPYHGP-----GQAHGLAFSVRPGIA-IPPSLLNM   92
VcUng    [18] YFQQTLQFVESQRQAGKVIYPPAKDVFNAFRFTEFGEVKVVILGQDPYHGP-----NQAHGLCFSVLPGVK-TPPSLVNI   92
CbUng    [21] YFQEILDFVKKERKAGKIIYPPQKDIFNALKLTPYEAIKVVILGQDPYHGP-----NQAHGLAFSVRPGVP-APPSLQNI   95
BsUng    [20] YYQELREMLKREYAE-QTIYPDSRDIFNALHYTSYDDVKVVILGQDPYHGP-----GQAQGLSFSVKPGVK-QPPSLKNI   93
MtUng    [23] QVAHMGQFLRAEIAAGRRYLPAGSNVLRAFTF-PFDNVRVLIVGQDPYPTP-----GHAVGLSFSVAPDVRPWPRSLANI   97
DrUng    [38] YFHELTDFLRQERKE-YTIYPPAPDVFNALRYTPLGEVKVLILGQDPYHGP-----NQAHGLSFSVRPGVR-VPPSLRNI  111
hUNG    [100] YFIKLMGFVAEERKH-YTVYPPPHQVFTWTQMCDIKDVKVVILGQDPYHGP-----NQAHGLCFSVQRPVP-PPPSLENI  173
cUNG     [97] YFKQLMSFVADERSR-HTVYPPADQVFSWTEMCDIQDVKVVILGQDPYHGP-----NQAHGLCFSVQKPVP-PPPSLVNI  170
lUNG    [160] AFIRIERFLDEEKEKGRVILPPAADIFNAFNSCPFRGLKVVLLGQDPYHDL-----HQAHGLCFSVLPEVP-LPPSLRNI  234
HSVUNG  [133] LTAHLLAEYNRRCQ-TEEVLPPREDVFSWTRYCTPDEVRVVIIGQDPYHHP-----GQAHGLAFSVRANVP-PPPSLRNV  206
EBVUNG   [46] KLAAVIACVRRLRT-QATVYPEEDMCMAWARFCDPSDIKVVILGQDPYHG------GQANGLAFSVAYGFP-VPPSLRNI  118
KSVUNG   [43] KLLSLLRQIRELRL-TTTVYPPQDKLMWWSHCCDPEDIKVVILGQDPYHK------GQATGLAFSVDPQCQ-VPPSLRSI  115
mvUNG   [145] YFPQLESKLSSYLEKRQRIVPYPELLFNTMNVLPPGKIKVVILGQDPYPGSCISGVPYAMGCSFSVPLNCP-VPKSLANI  224

SaUng         YKELADDI-GCVR-QTPHLQDWAREGVLLLNTVLTVRQGEANSHR-DIGWETFTDEIIKAVSDY-KEHVVFILWGKPAQQ  163
EcUng         YKELENTIPGFTRPNHGYLESWARQGVLLLNTVLTVRAGQAHSHA-SLGWETFTDKVISLINQH-REGVVFLLWGSHAQK  170
VcUng         YKELAQDIPGFQIPPHGYLQSWAQQGVLLLNTVLTVEQGMAHSHA-NTGWETFTDRVIDALNQH-RNGLIFLLWGSHAQK  170
CbUng         FKELHADL-GVSIPSHGFLEKWAKQGVLLLNAALTVEAGKPQSHA-NIGWHRFTDKVIESLNDH-PEGIVFLLWGSYAQK  172
BsUng         FLELQQDI-GCSIPNHGSLVSWAKQGVLLLNTVLTVRRGQANSHK-GKGWERLTDRIIDVLSER-ERPVIFILWGRHAQM  170
MtUng         FDEYTADL-GYPLPSNGDLTPWAQRGVLLLNRVLTVRPSNPASHR-GKGWEAVTECAIRALAAR-AAPLVAILWGRDAST  174
DrUng         YKELTEDIPGFVAPKHGYLRSWAEQGVLLLNAVLTVRAGQANSHQ-GKGWEHFTDAVIKAVNAK-EERVVFILWGSYARK  189
hUNG          YKELSTDIEDFVHPGHGDLSGWAKQGVLLLNAVLTVRAHQANSHK-ERGWEQFTDAVVSWLNQN-SNGLVFLLWGSYAQK  251
cUNG          YKELCTDIDGFKHPGHGDLSGWAKQGVLLLNAVLTVRAHQANSHK-DRGWETFTDAVIKWLSVN-REGVVFLLWGSYAHK  248
lUNG          YKELTTDIAGFQAPKHGYLQSWSEQGMLMLNATLTVEAHKANSHSKTSGWAAFTDAVIQHLSQHHPNRLVFLLWGGYAQQ  314
HSVUNG        LAAVKNCYPEARMSGHGCLEKWARDGVLLLNTTLTVKRGAAASHS-RIGWDRFVGGVIRRLAAR-RPGLVFMLWGTHAQN  284
EBVUNG        YAELHRSLPEFSPPDHGCLDAWASQGVLLLNTILTVQKGKPGSHA-DIGWAWFTDHVISLLSER-LKACVFMLWGAKAGD  196
KSVUNG        FRELEASVPNFSTPSHGCLDSWARQGVLLLNTVLTVEKGRAGSHE-GLGWDWFTSFIISSISSK-LEHCVFLLWGRKAID  193
mvUNG         YTNLIKFNHMRKAPKHGCLASWILQGTFMINSAFTTVLNESGVHA--RTWESFTADLIDYLTDN-YDDLIFVAWGAHAHK  301
                                   ▼
SaUng         KIKLIDTSKHCIIKSVHPSPLS-----------------AYRGFFGSKPYSKANAYLESVGKSPINW [5]    218
EcUng         KGAIIDKQRHHVLKAPHPSPLS-----------------AHRGFFGCNHFVLANQWLEQRGETPIDW [9]    229
VcUng         KGQMIDRQRHHVLMAPHPSPLS-----------------AHRGFLGCRHFSKTNQLLQAQGIAPINW [6]    226
CbUng         KSQLITNLRHRILKAPHPSPLS-----------------AARGFLGCRHFSKANQLLHEMGRGEIDW [7]    229
BsUng         KKERIDTSKHFIIESTHPSPFS-----------------ARNGFFGSRPFSRANAYLEKMGEAPIDW [5]    225
MtUng         LKPMLAAGNCVAIESPHPSPLS-----------------ASRGFFGSRPFSRANELLVGMGAEPIDW [3]    227
DrUng         KKKLITGKNHVVIESGHPSPLS-----------------E-QYFFGTRPFSKTNEALEKAGRGPVEW [9]    247
hUNG          KGSAIDRKRHHVLQTAHPSPLS-----------------VYRGFFGCRHFSKTNELLQKSGKKPIDW [3]    304
cUNG          KGATIDRKRHHVLQAVHPSPLS-----------------AHRGFLGCKHFSKANGLLKLSGTEPINW [3]    301
lUNG          KKRLIDASRHVVLENVHPSPLS-----------------ANRGWFGCRCFSACNEALQRMSHLPMYW [9]    373
HSVUNG        AIR-PDPRVHCVLKFSHPSPLS-----------------K-VPFGTCQHFLVANRYLETRSISPIDW [2]    334
EBVUNG        KASLINSKKHLVLTSQHPSPLAQNST----------RKSAQQKFLGNNHFVLANNFLREKGLGEIDW [2]    255
KSVUNG        RTPLINAQKHLVLTAQHPSPLASLGG----------RHSRWPRFQGCNHFNLANDYLTRHRRETVDW [5]    255
mvUNG         LCQRVDPKKHYIITSSHPSPYSVSNTMTSMSYGPNPKKVTYPSFNSVDHFGKINEHLKSRNKKPIFW [2]    370
```

Figure S3

Predicted Local Distance Difference Test (pLDDT) values for AlphaFold 3-generated models of SaUNG. pLDDT is a per-atom confidence estimate on a 0–100 scale, where a higher value indicates higher confidence. A, free wild-type SaUNG; B, free SaUNG H180A; C, free SaUNG D59A/H180A; D, wild-type SaUNG–DNA, the protein part of the complex; E, SaUNG H180A–DNA, the protein part of the complex; F, SaUNG D59A H180A–DNA, the protein part of the complex; G, wild-type SaUNG–DNA, the DNA part of the complex; H, SaUNG H180A–DNA, the DNA part of the complex; I, SaUNG D59A H180A–DNA, the DNA part of the complex. Each dot shows pLDDT for the Cα (protein) or C1′ (DNA) atoms at the respective position in one model; most of the dots overlap due to similar pLDDT values. The dashed lines show the threshold levels of structure prediction confidence; the confidence interpretation is presented in Panel A. The interface predicted template modeling (ipTM) score for all complexes was 0.96, with protein–DNA chain pair ipTMs in the 0.94–0.97 range. The ipTM score ranges from 0 to 1, with values >0.8 indicating a confidently predicted interaction.

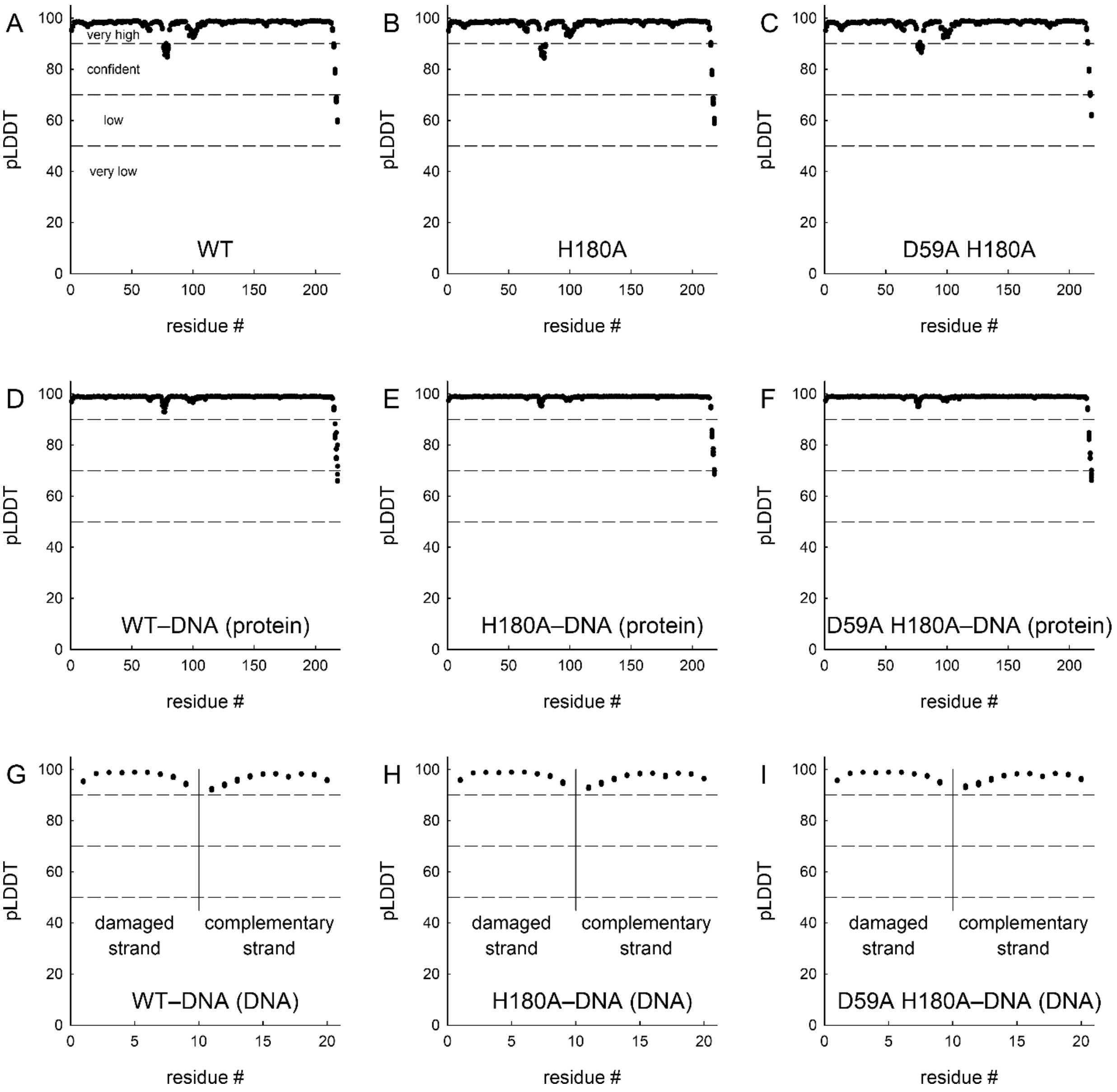

Figure S4

Reconstitution of the *S. aureus* uracil base excision repair pathway. The assay was performed using purified proteins with an increased amount of SaNfo (150 nM). Asterisks denote the 5′ -terminal fluorescent label.

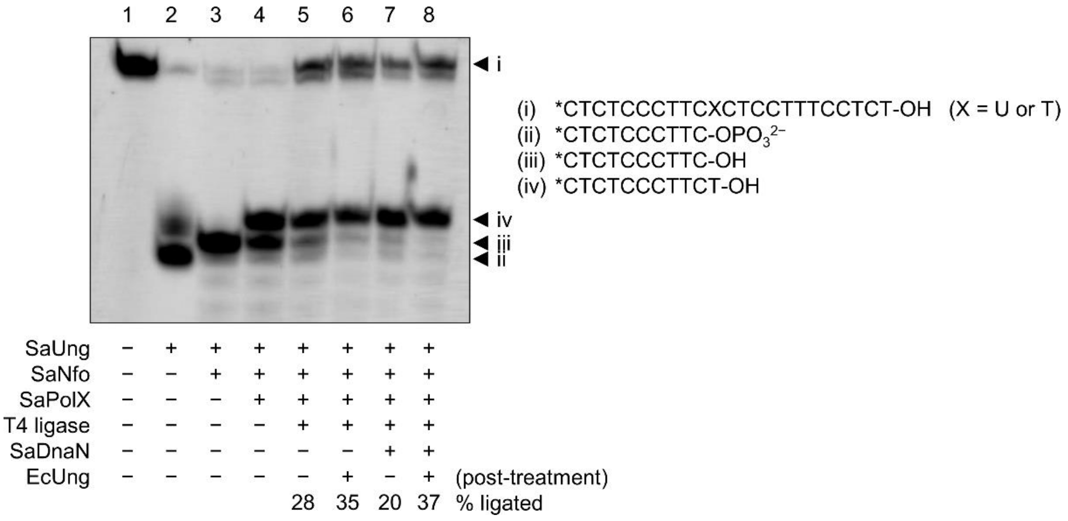

Supplement: Supplementary file 1 [file pathogens-15-01012-s001.zip › pathogens-4515333-supplementary.pdf]
